# CBCT dual-center investigation of relationship between the inferior alveolar canal and the lingual mandibular depression of the submandibular salivary glands

**DOI:** 10.1038/s41598-025-26973-3

**Published:** 2025-12-02

**Authors:** Maha Jamal Abbas, Ali R. Al-Khatib, Nuhad A. Hassan, Abeer A. Aljoujou, Alaa ALhomsi, Majd Walid ALsalim

**Affiliations:** 1 Orthodontics, Prevention and Pedodontics Department, College of Dentistry, AL- Mustansiriyah University, Baghdad, Iraq; 2 Department of Pedodontics, Orthodontics and Preventive Dentistry, College of Dentistry, University of Mosul, Mosul, Iraq; 3 Oral Medicine Department, College of Dentistry, AL- Mustansiriyah University, Baghdad, Iraq; 4 Department of Oral Medicine, Faculty of Dental Medicine, Damascus University, Damascus, Syrian Arab Republic; 5 Ministry of Health, Damascus, Syrian Arab Republic

**Keywords:** Salivary glands, Dental implants, Mandible, Cone beam computed tomography, Inferior alveolar canal, Anatomy, Diseases, Health care, Medical research

## Abstract

This study meticulously examined the depth of the lingual concavity (LCD) in the posterior part of the mandible and its relationship with the inferior alveolar canal (IAC), using cone beam computed tomography (CBCT). The results were then compared across different sides, sexes, age groups, and ethnic backgrounds. A cross-sectional study was conducted, analyzing 200 CBCT scans, with 100 scans from Iraq and 100 from Syria. The location of the deepest LC was carefully examined in relation to the IAC and categorized into three groups: above the canal, at the same level as the canal, and below the canal. The statistical analyses were performed with thoroughness and precision using SPSS version 25 (IBM Corp., Armonk, NY, USA). The study revealed a significant relationship between the deepest LCD and the sides. Notably, the deepest LCD showed significant differences with age groups on the right side (*p* 0.026). The LC was found to be deeper in Syria (2.05 mm) for both sides, compared to Iraq, where it was 1.8 mm on the right and 1.6 mm on the left. The left and right sides of the LC were found to have different depths, with the right side being deeper than the left. The deepest part of the fossa was discovered to be at the level of the mandibular canal on the left side.

## Introduction

In clinical practice, dental implant (DI) therapy is routinely used to replace missing teeth^1^. It is crucial to meticulously assess the bone placement before inserting DIs. The shape of the bone and the way implants interact with vital structures are pivotal for implant planning^2,3^. Any oversight in planning for implants can result in serious complications such as bone perforation, inflammation, infection, a broken jaw, and implant loss^4^.

Lingual concavity (LC) refers to a depression on the medial surface of the mandible, typically situated in the molar region^5^. This concavity is primarily formed by the compressive force exerted by the submandibular salivary gland on the bony cortex. Submandibular fossae are the sites of the majority of perforations that are linked to implant placement^6^.

LC and inferior alveolar canal (IAC) operate as limiting structures in determining the length of the DI fixture. The risk factor for lingual plate perforation is LC, which can result in a variety of complications, including local edema and life-threatening events, if improper surgical insertion of a DI is performed^7,8^.

Damage to the IAC can cause permanent neurosensory problems, such as teeth losing their vitality and the chin being numb in the afflicted quadrant. The lingual nerve is damaged by perforation of the cortex as it extends from the lingual aspect of the mandible, which contributes to the persistent infection of the posterior mandible. The patient experiences severe life-threatening problems, such as obstruction of the upper airway, as a result of the infection’s dissemination to the parapharyngeal and retropharyngeal compartments^9,10^.

The panoramic radiograph can assess the length of the implant; however, it is unable to provide information on the buccolingual dimensions. In order to reduce complications, it is advisable to employ three-dimensional (3D) radiological techniques. One such technique is CBCT. Cross-sectional analysis and 3D reconstruction are facilitated by CBCT, which boasts a higher degree of accuracy and a lower radiation dose^11,12^.

Understanding the intricacies of LC in the posterior mandible is a pivotal aspect of dental implantology. Previous studies by Sun et al.^13^ and ALQutaibi et al.^14^ have highlighted the potential challenges posed by LC in the posterior mandible during implant surgery. Notably, there is no prior study that compares two Arab countries. As a result, this investigation utilized CBCT images to delve into the depth of the LC at the posterior portion of the mandible and its relationship with the IAC. It also explored how this depth varies with age, sex, and ethnicity. The study’s null hypothesis suggested that there is no significant difference in the depth of the lingual concavity across different sides, sexes, age groups, or populations.

## Methods

### Study population

The CBCT images of 200 individuals (100 Iraqi and 100 Syrian), 112 males and 88 females, were collected from patients who underwent CBCT for any other dental purpose. The minimum sample size was determined to be 60 patients per group (the estimated effect size of d (0.604)) using the G*Power 3.1.9.7 tool, along with data from the pilot study.

All subjects signed informed consent documents, and the study adhered to the principles of the Helsinki Declaration. The Ethics Committee of Al-Mustansiriyah University/College of Dentistry approved the study (MUOMED06).

#### Inclusion criteria

Adult patients (≥ 18 years) who have high-quality CBCT scans of the dentate posterior mandible were included.

#### Exclusion criteria

Patients with congenital syndromes, a history of trauma, any pathological conditions, exostosis, and those who had posterior mandibular surgery. Additionally, excluded CBCT scans lack clarity and have the potential for containing implants or other foreign bodies that could introduce artifacts.

### CBCT set

“The data was collected using a Kodak 9500 Cone Beam 3D System (Carestream Health Inc., Croissy-Beaubourg, Marne la Vallée, France)”. The exposure time was 10.8 s, with settings of 90 kV and 10 mA; the voxel size was 0.3 mm. “All images were analyzed with CS 3D Imaging software version 3.5.7 (Carestream Health Inc., Croissy-Beaubourg, Marne la Vallée, France) “.

### Measurements

In each scan, the mandible’s two sides are examined. The mandible is reconstructed using panoramic, sagittal, axial, and cross-sectional images. IAC is recognized. Every cross-sectional image between the first premolar and second molar region is subjected to measurements. The scan that demonstrated the deepest LC is measured in cross-sections at 1 mm intervals, with a thickness of 1 mm from the mesial of the first premolar to the distal of the second molar.

**Fig. 1:**
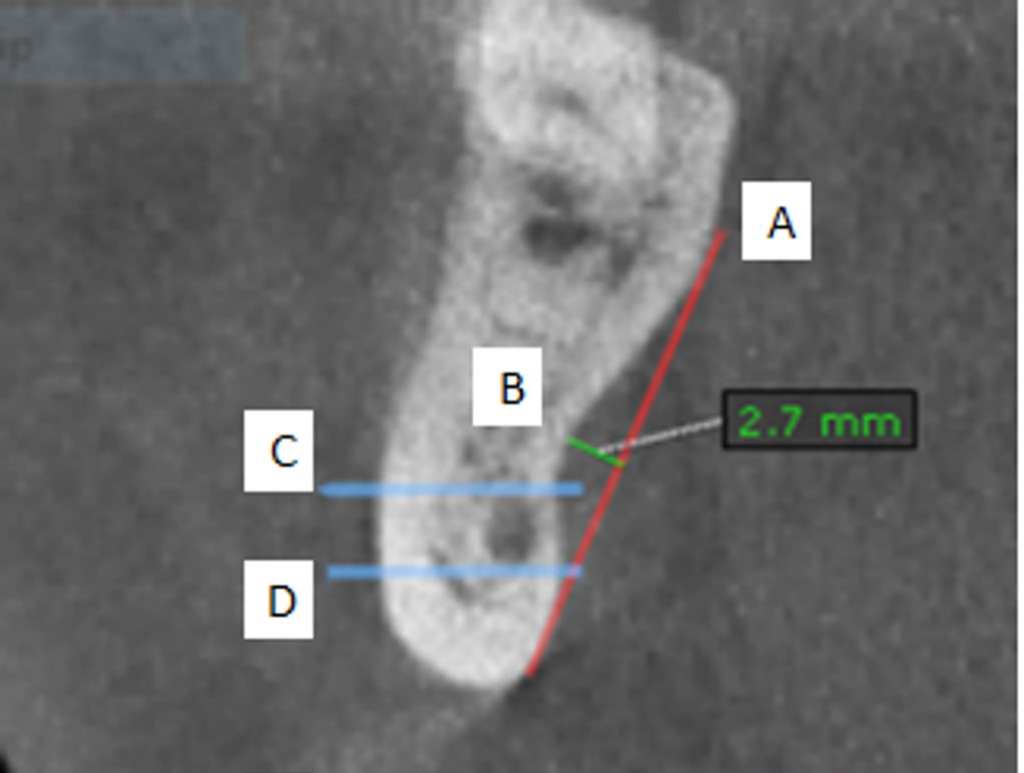
illustrates the depth measurements of the lingual concavity as observed in CBCT scans, as well as the position of the inferior alveolar canal. Line A represents the measurement from the upper to the lower bony prominences of the concavity. Line B is drawn perpendicular to Line A, starting from the deepest point of the lingual concavity, to represent the measured distance. Lines C and D denote the superior and inferior borders of the inferior alveolar canal.

**Fig. 2:**
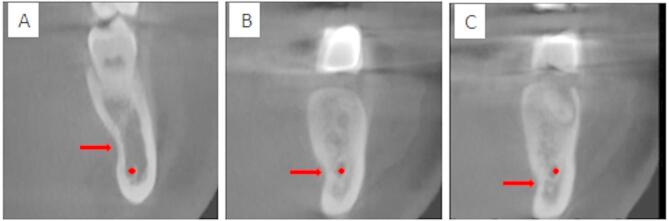
The relationship between the depth of the deepest lingual concavity: above the inferior alveolar canal (**A**), at the level of the inferior alveolar canal (**B**), and below the level of the inferior alveolar canal (**C**).

Next, draw a line (line A) connecting the upper and lower bony prominences of LC. Then, draw a line (line B) that is perpendicular to line A, extending from the deepest point of the LC, and measure the distance. Additionally, draw lines C and D to indicate the level of the IAC. The position of the deepest LC is then compared to the IAC to determine its location: whether it is above line C, between lines C and D, or below line D, as illustrated in Figs. 1 and 2^15^.

The examiners were trained to utilize only cross-sectional images for measuring the deepest LC and its relationship to the IAC. However, they had the flexibility to navigate through the 1 mm intervals of cross-sectional images to assess the deepest LC.

The data collection process involved repeating measurements twice to calculate the mean as the final value. Examiners were standardized to identify the location of the LC in relation to the IAC by analyzing 10 CBCT scans. The intra-observer and inter-observer reliability between the two examiners demonstrated a high level of agreement (Intraclass Correlation Coefficient (ICC) values ranged from 0.85 to 0.92; kappa > 0.89; 95% confidence intervals).

### Statistical analysis

The statistical analyses were performed with thoroughness and precision using SPSS version 25 (IBM Corp., Armonk, NY, USA). Normality was evaluated with the Shapiro–Wilk test, which indicated a non-normal distribution, ensuring the validity of the results.

The deepest LCD was compared between sides, sexes, countries, and age groups, with a P value < 0.05 denoting statistical significance (Mann-Whitney U test, Chi-square test, Kruskal-Wallis test). The correlation coefficient shows the relationship between LCD, the sides, and the age groups (Spearman correlation test).

## Results

The patients’ ages ranged from 18 to 60 years; mean (35.83); divided into three groups (18–29, 30–45, and 46–60 years)^15^, with approximately 40.5% of them falling between 18 and 29 years old. As shown in Table 1, the findings indicate that the depth of the fossa on the right side of the mandible was greater than that on the left side (*P* < 0.001).

**Table 1:** Lingual concavity depth (mm) based on side.

Variable	Side	Mean	Median	Interquartile	*P*-value
LCD ^a^	Right	2.023	2	0.8	0.000^*^
	Left	1.880	1.9	0.8	

Mann-Whitney U test; 95% confidence; ^a^ LCD: lingual concavity depth; * significant.

In Table 2, the depth of the fossa on both sides showed no significant association with sex. However, it was greater in Syrian patients compared to Iraqi patients, with P-values of 0.006 for the right side and 0.003 for the left side.

**Table 2:** Lingual concavity depth (mm) in different study groups based on side.

	Group	Mean	Median	Interquartile	*P*-value
RLCD^a^	Male	2.321	2	0.8	0.722
	Female	2.108	1.9	0.8	
LLCD^b^	Male	2.147	1.9	1.1	0.761
	Female	1.985	1.9	0.7	
RLCD	Iraq	1.884	1.8	0.8	0.006*
	Syrian	2.18	2.05	0.8	
LLCD	Iraq	1.768	1.6	0.9	0.003*
	Syrian	2.04	2.05	0.6	

Mann-Whitney U test; 95% confidence; ^a^ RLCD: right lingual concavity depth; ^b^ LLCD: left lingual concavity depth; * significant.

The depth of the fossa on the right side showed a significant relationship with age, specifically being lower in individuals under 29 years old (P 0.026) (Table 3).

**Table 3:** Lingual concavity depth (mm) among different age groups based on side.

Side	Age-group/Years + Mean	Median	Interquartile	*P*-value
RLCD ^a^	18–29 (22.8)	1.9	0.9	0.026^*^
	30–45 (37.43)	2	1	
	46–60 (53.77)	2	0.9	
LLCD ^b^	18–29 (22.8)	1.9	0.9	0.607
	30–45 (37.43)	1.8	0.9	
	46–60 (53.77)	1.9	0.7	

Kruskal-Wallis test; ^a^ RLCD: right lingual concavity depth; ^b^ LLCD: left lingual concavity depth; * significant.

According to Table 4, the deepest part of the fossa on the right side, located above and to the left, was at the level of the mandibular canal. There were no significant differences among patients in terms of sex and ethnicity. Additionally, while concavity was primarily observed above the location on the right side and at the level of the canal on the left, no significant disparities were found between the fossa depth and age groups.

**Table 4:** Relation of the deepest lingual concavity to the location of the inferior alveolar Canal in different study groups.

Group	Side	Variable	Above	Level	Below	*P*-value
Country	RLCD^a^	Iraq	33.5%	16.0%	0.5%	0.215
		Syria	27.5%	22.0%	0.5%	
	LLCD^b^	Iraq	19.0%	27.0%	4.0%	0.730
		Syria	17.5%	29.5%	3.0%	
Gender	RLCD	Male	24.5%	19%	0.5%	0.393
		Female	36.5%	19%	0.5%	
	LLCD	Male	14%	26.5%	3.5%	0.464
		Female	22.5%	30%	3.5%	
Age-groups	RLCD	18–29	28.0%	12.0%	0.5%	0.073
		30–45	18.5%	10.0%	0.0%	
		46–60	14.5%	16.0%	0.5%	
	LLCD	18–29	17.0%	19.5%	4.0%	0.319
		30–45	10.0%	17.0%	1.5%	
		46–60	9.5%	20.0%	1.5%	

Chi-square test; ^a^ RLCD: right lingual concavity depth; ^b^LLCD: left lingual concavity depth.

The correlation coefficient between fossa depth and age groups was found to be positive and significant on the right side. This indicates that as age increases, the depth of the fossa also increases (P 0.007) (Table 5).

**Table 5:** Correlation between deepest lingual concavity depth (mm) of both side with age group and between them.

Variables	Correlation Coefficient	Sig. (2-tailed)
Age-group &RLCD ^a^	0.192^**^	0.007*
Age-group &LLCD ^b^	0.009	0.897
RLCD&LLCD	0.596^**^	0.000*

Spearman correlation test; ^a^ RLCD: right lingual concavity depth; ^b^ LLCD: left lingual concavity depth; * significant.

## Discussion

Concavities are very important for the success and longevity of DIs in the mandibular arch. To make sure that implants are put in safely and that patients have good dental health, implantologists need to know how deep the implants should be. If you overlook concavities, you may end up with an incorrect implant angle and insufficient prosthetic support. Additionally, perforating the lingual plate can result in severe complications, including bleeding, inflammation, and infection, as well as life-threatening situations like airway obstruction^16,17^.

2D imaging techniques, such as panoramic images, are insufficient for accurately assessing the LC. In contrast, 3D imaging, particularly CBCT, offers a comprehensive radiographic evaluation. CBCT cross-sections provide the highest spatial resolution along with the lowest cost and radiation exposure for the assessment of bony structures; it is also recommended for immediate implantation^18,19^. This comprehensive nature of 3D imaging should reassure implantologists about its effectiveness in their practice.

Based on the research by Alqahtani et al.^20^, which involved an investigation of 165 CBCT images, it is important to check the mandibular LC when placing implants to avoid accidentally damaging the lingual plate. This study provided an accurate assessment of the LCs, leading to the conclusion that this step is crucial in implant placement^20^.

Chaubal et al.^15^ looked at 384 CBCT scans and found that because of differences in depth and the chance of penetration, decisions based only on LCD and its connection to the IAC should lead to assessing the implant fixture site individually for each case during pre-operative planning for DI surgery.

The results of this study indicated a discrepancy in LCD between the two sides. In line with this finding, Khodabakhshian et al.^21^ reported that the mean depth of the fossa on the left side (1.66 ± 0.46 mm)was statistically less than that on the right side (1.79 ± 0.48 mm), based on an analysis of CBCT images from 59 men and 66 women. Additionally, Tan et al.^22^ noted a significant difference in LCD between the two sides, linking this difference to the varying sizes of the submandibular gland on each side.

The results indicated no discrepancies in LCD based on sex. Similarly, other studies have not found significant differences in LCD between men and women^6,11^, which aligns with our findings. However, Khodabakhshian et al.^21^ and Şahan et al.^23^ reported that men exhibited much deeper average LCDs on both sides. Additionally, Ramaswamy et al.^24^ and Bayrak et al.^1^noted that the average LCD was greater on the right side for both men and women. These variations among studies could potentially be attributed to ethnic differences.

Recently, Sadik et al.^25^ and Levingston et al.^26^ identified significant disparities in sex and laterality within Turkish and Indian populations, respectively. It is crucial to radiologically assess these anatomical structures prior to surgery to prevent complications^25,26^. These findings bolster our assertion that variations in LC are clinically significant and should be meticulously evaluated during implant planning, considering ethnic differences and potential side effects. Parnia et al.^11^and Nickenig et al.^9^ previously reported that when the LCD exceeds 2 mm, the rates of perforation in the lingual cortex and associated complications increase.

The results indicated a difference in LCD between the two study countries. Chaubal et al.^15^ reported an ethnic variation that aligns with these findings. Almarghlani et al.^27^ emphasized the significance of studying LC across various ethnicities and races.

Regarding age groups, Khodabakhshian et al. found that the LCD on the right side was significantly lower in patients under 36 years old (*P* = 0.005), while this study determined a similar finding for those under 29 years old^21^. In contrast, Bayrak et al.‘s investigation produced conflicting results^1^. Their study categorized patients into three distinct age groups: under 20, 20–44, and over 45. This discrepancy may stem from varying age classifications and demographic differences related to the studies.

This finding aligns with the study by Kamburoğlu et al.^28^ regarding the right side, which revealed a correlation between the LC indicators and the participants’ age.

The present study identified that the deepest part of the fossa on both sides is positioned above on the right side and adjacent to the canal on the left side. Khodabakhshian et al.^21^ noted that the deepest part of the fossa is located next to the mandibular canal, and they found that the left side exhibits more parallel relationships compared to the right side, which aligns with our findings. Haghanifar et al.^4^, on the other hand, reported that the deepest part lies above the canal on both sides and in all sexes. Şahan et al.^23^, Sadik et al.^25^ (in Turkish), and Ramaswamy et al.^24^ (in Indian) conducted studies indicating that the IAC was most often found inferiorly relative to the deepest point of the LC. This variability can be explained by the differing measurement protocols used in various studies, as well as by ethnic diversity and variations in sample sizes. Overall, the findings indicate the rejection of the null hypothesis, as statistically significant differences were identified in the LCD across various sides, age groups (specifically the right side), and populations (*p* < 0.05).

### Limitation

The study was limited to dentate subjects, had a restricted sample size, lacked functional assessments, did not evaluate salivary gland volume due to the limitations of CBCT, and was specific to a certain population. These factors may impact the generalizability of the results.

## Conclusion

The influence of the sides on the LCD is notable, with the right side exhibiting a more significant effect than the left. These variations may stem from anatomical asymmetries, masticatory forces, and individual differences. Syria shows higher measurements indicative of LCD on both sides compared to Iraq, reflecting ethnic differences. The deepest part of the fossa is generally found above the right side and at the level of the canal on the left, taking into account factors such as sex, ethnicity, and age groups. There is a moderate correlation between LCDs on both sides and age groups. These findings prove the value of performing site-specific CBCT preoperative evaluations in implant dentistry, resulting in improved patient outcomes.

### Future recommendation

Future studies should involve larger, multi-center, multi-ethnic samples and consider different dentate statuses and salivary gland volume evaluations using alternative modalities, as CBCT has limitations in assessing soft tissues.

## Data Availability

The datasets generated during and/or analysed during the current study are available from the corresponding author on reasonable request.
